# Supplementary material for: Endothelial TRPV4/Cx43 Signaling Complex Regulates Vasomotor Tone in Resistance Arteries
Source: bioRxiv. 2024 Jul 25:2024.07.25.604930. Preprint. [Version 1] doi: 10.1101/2024.07.25.604930 (PMC11291137; doi:10.1101/2024.07.25.604930)
Supplement: Supplement 1 — Supplemental Figure 1: Validation of Cx45 KO cell line. A) Sanger sequencing of Cx45 from Wild-type HeLa cells (top) and the clone # 18 used in this work. The mutation induced by CRISPR (Synthego) induced the deletion of 2 nucleotides causing a frameshift in the Cx45 sequence (asterisk). The guide sequence used for guide RNA synthesis is shown as a horizontal black line. The vertical dotted line represents the cut site. B) Western blot of HeLa lysates from 4 different monoclonal populations and two different batches of Wild-type HeLa cells. Clone 18 was transiently transfected with rat Cx45 as a positive control for the Cx45 antibody and to demonstrate transfection efficiency in this clone. Lysates from Xenopus laevis oocytes were also used as control. Oocytes were injected with RNA for Cx45 and cell lysates were obtained 2 days after RNA injection. Non-injected oocytes were used as the negative control. C) Summary of ICE analysis. The predicted KO scores correlates with the signal for Cx45 detected by Western blot. Supplemental Figure 2. TRPV4 controls Cx43 hemichannel activity in a heterologous expression system. Activation of Cx43 hemichannels was evaluated by assessing ethidium uptake. Connexin-free HeLa cells were transfected with human EGFP-tagged alone or in combination with human TRPV4. Dye uptake was evaluated in basal conditions and in the presence of 1 μM GSK under constant superfusion (See Methods). (A) Representative time courses of ethidium uptake. Each group correspond to the average of several GFP-positive cells (n=22–112) from the same coverslip. (B) Quantification of the ethidium uptake rate. Statistical comparisons between groups were performed using one-way ANOVA and Tukey post hoc test. Supplemental Figure 3: TRPV4 decreases Cx43 expression. A) Representative images of the EGFP signal in cells transfected with EGFP alone or EGFP + TRPV4. Images were taken with the same exposure time (500 ms) for comparison. The quantification of EGFP signal inte [file media-1.pdf]

A

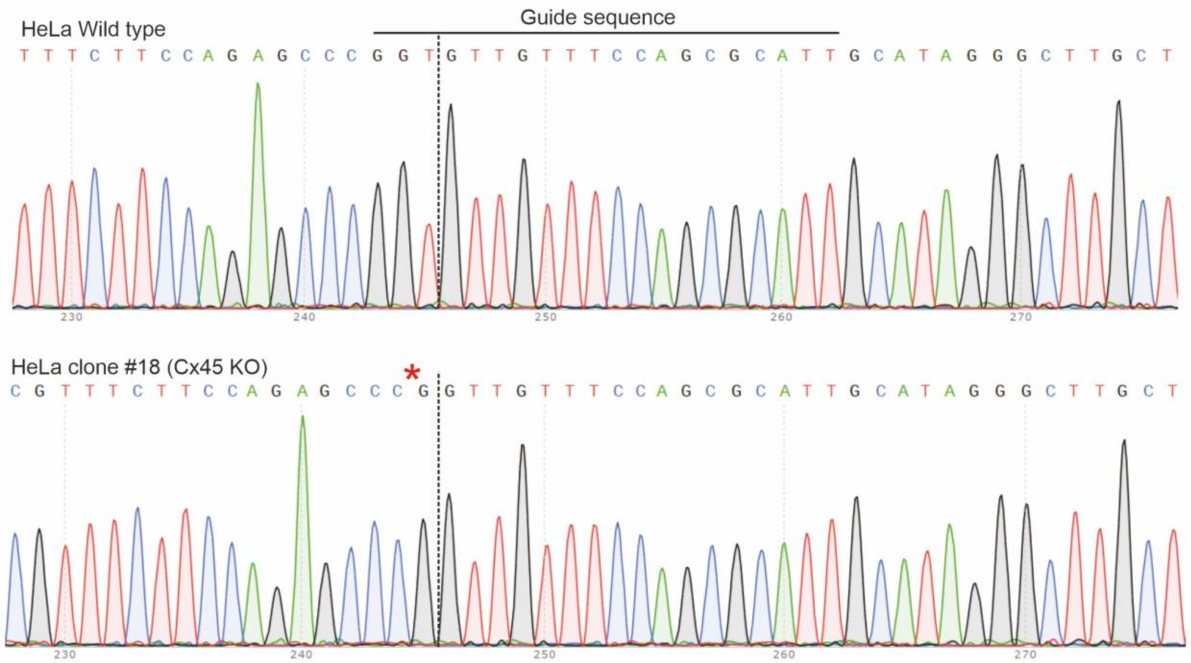

B

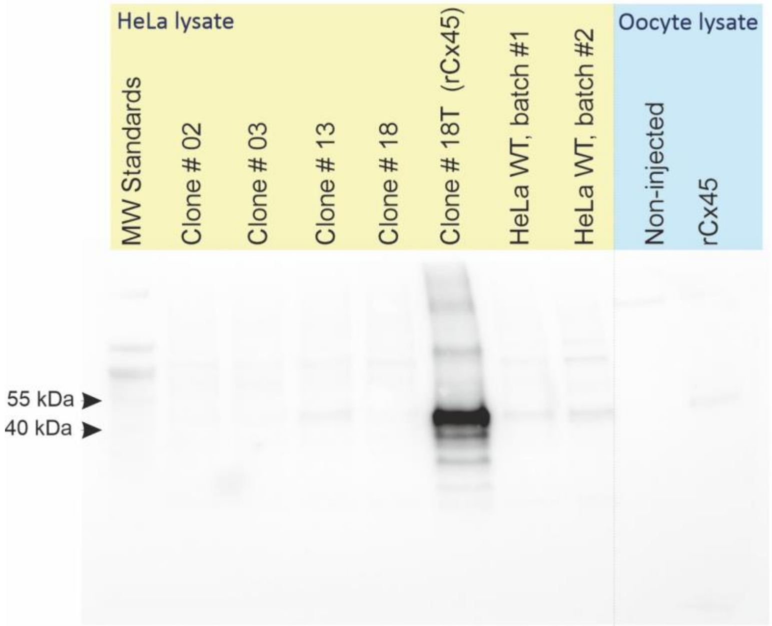

C

	KO score by ICE analysis	Western blot signal
Clone # 02	99	No
Clone # 03	99	No
Clone # 13	0	Yes
Clone # 18	100	No
Wild type pool	0	Yes

Supplemental Figure 1

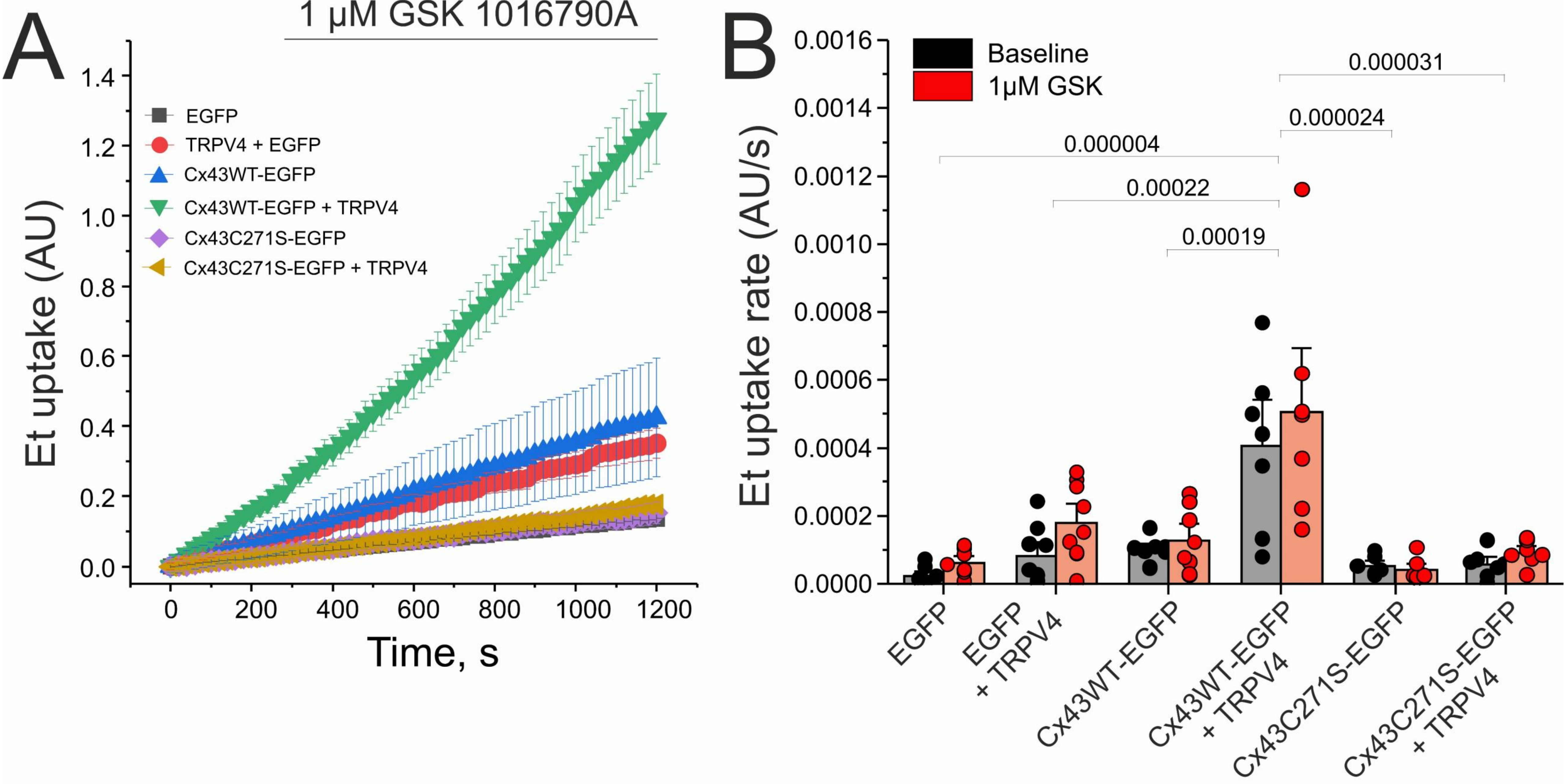

Supplemental Figure 2

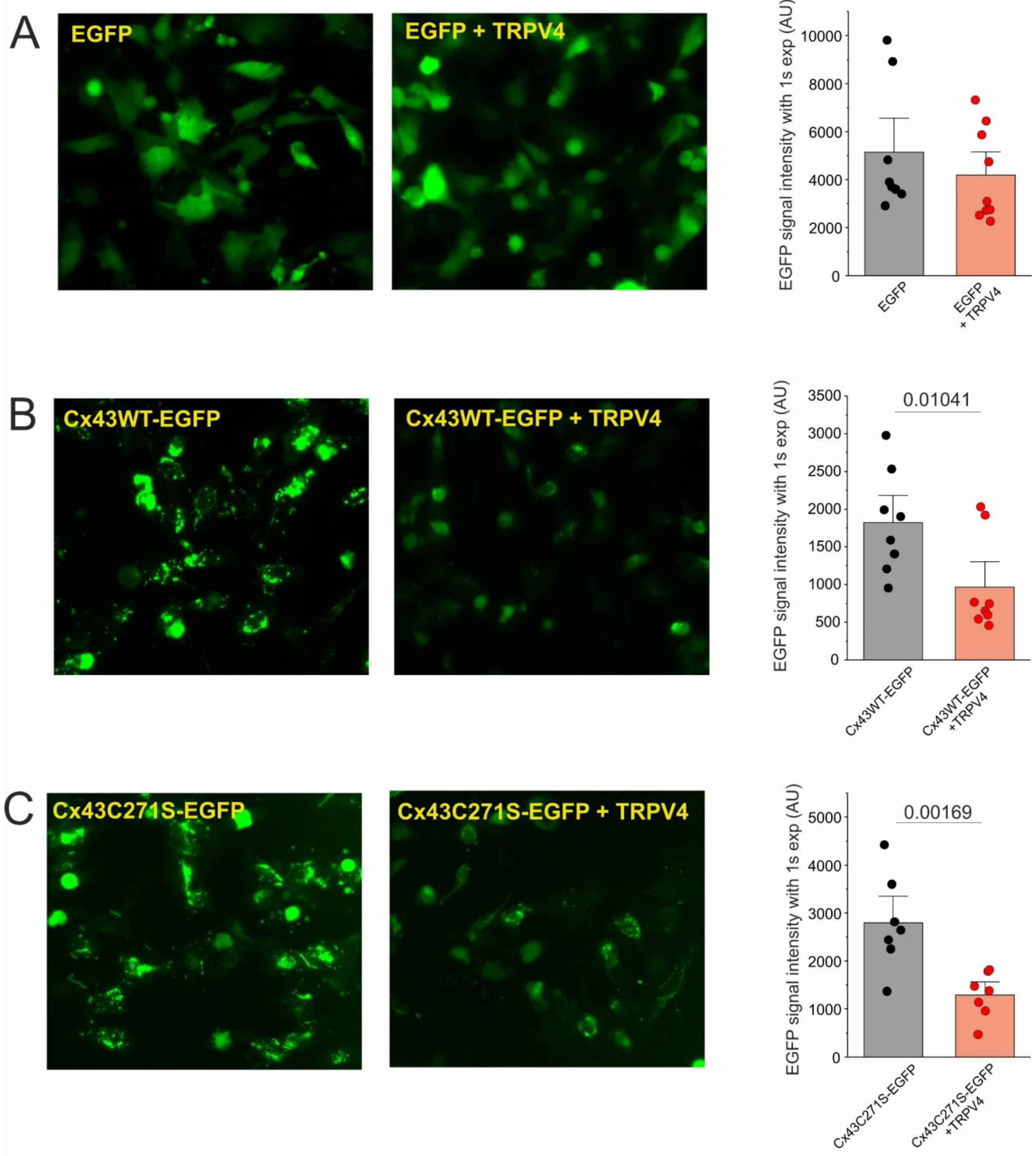

Supplemental Figure 3

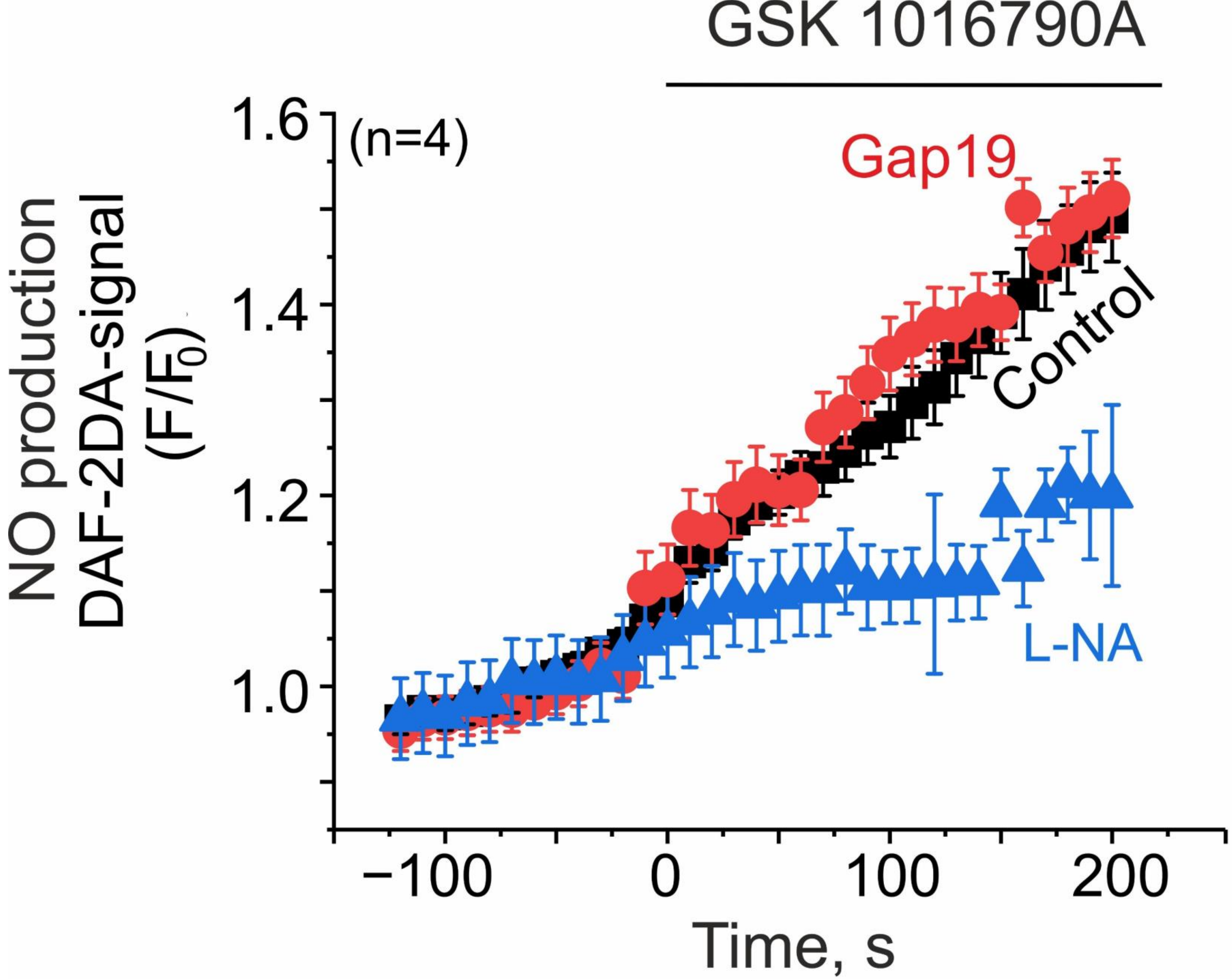

Supplemental Figure 4

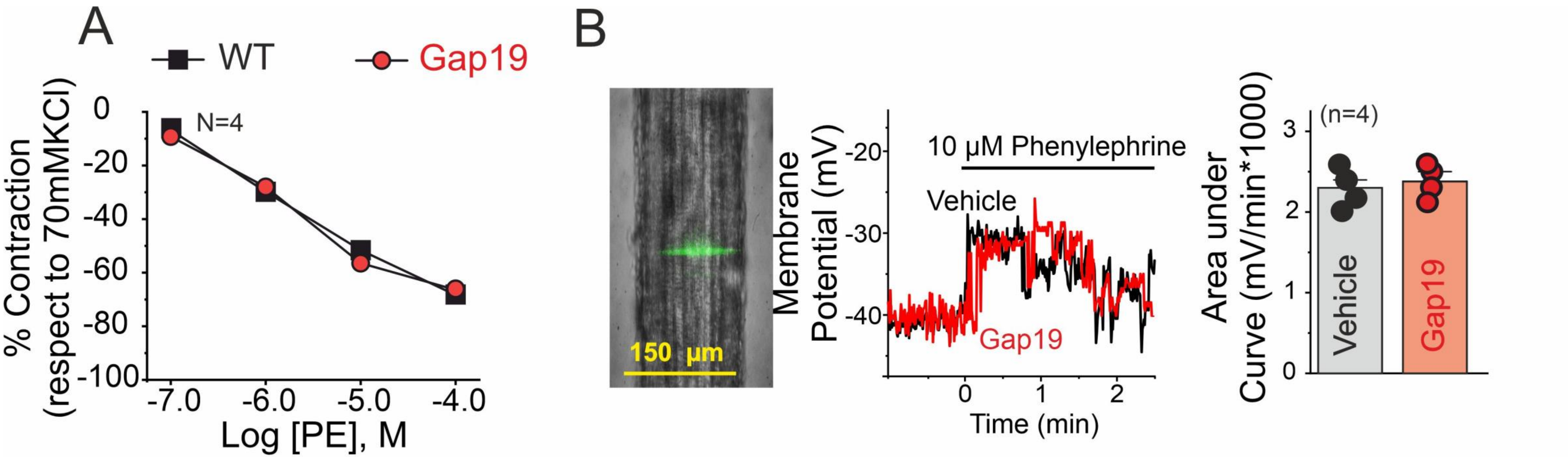

Supplemental Figure 5

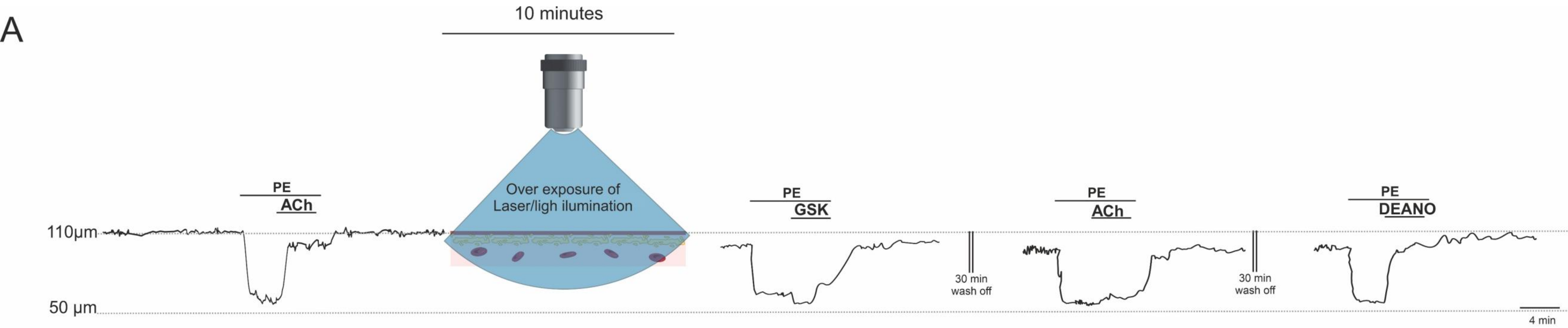

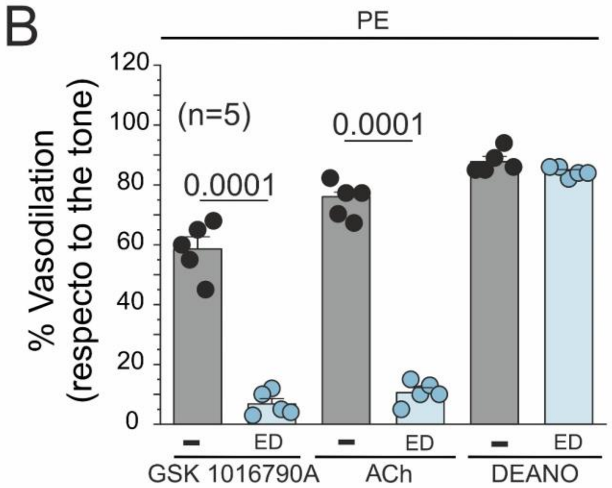

Supplemental Figure 6

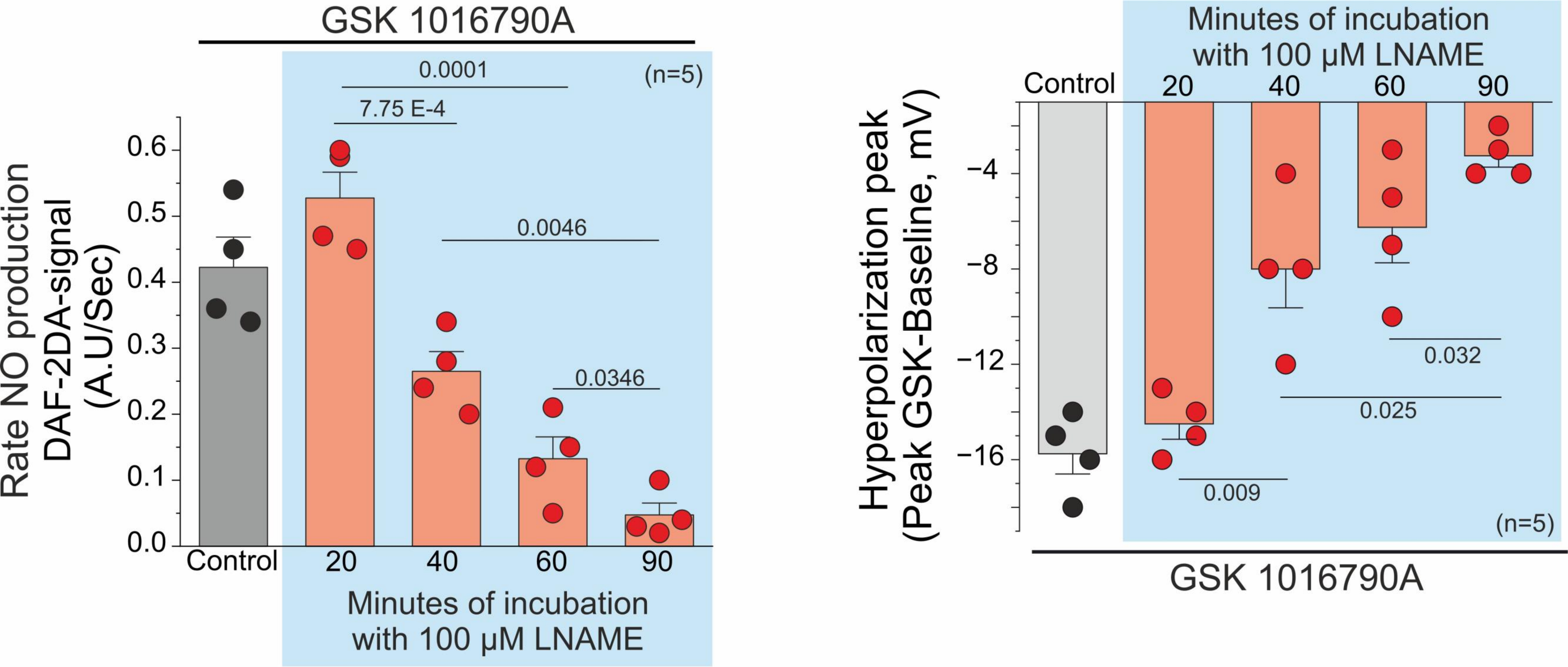

Supplemental Figure 7

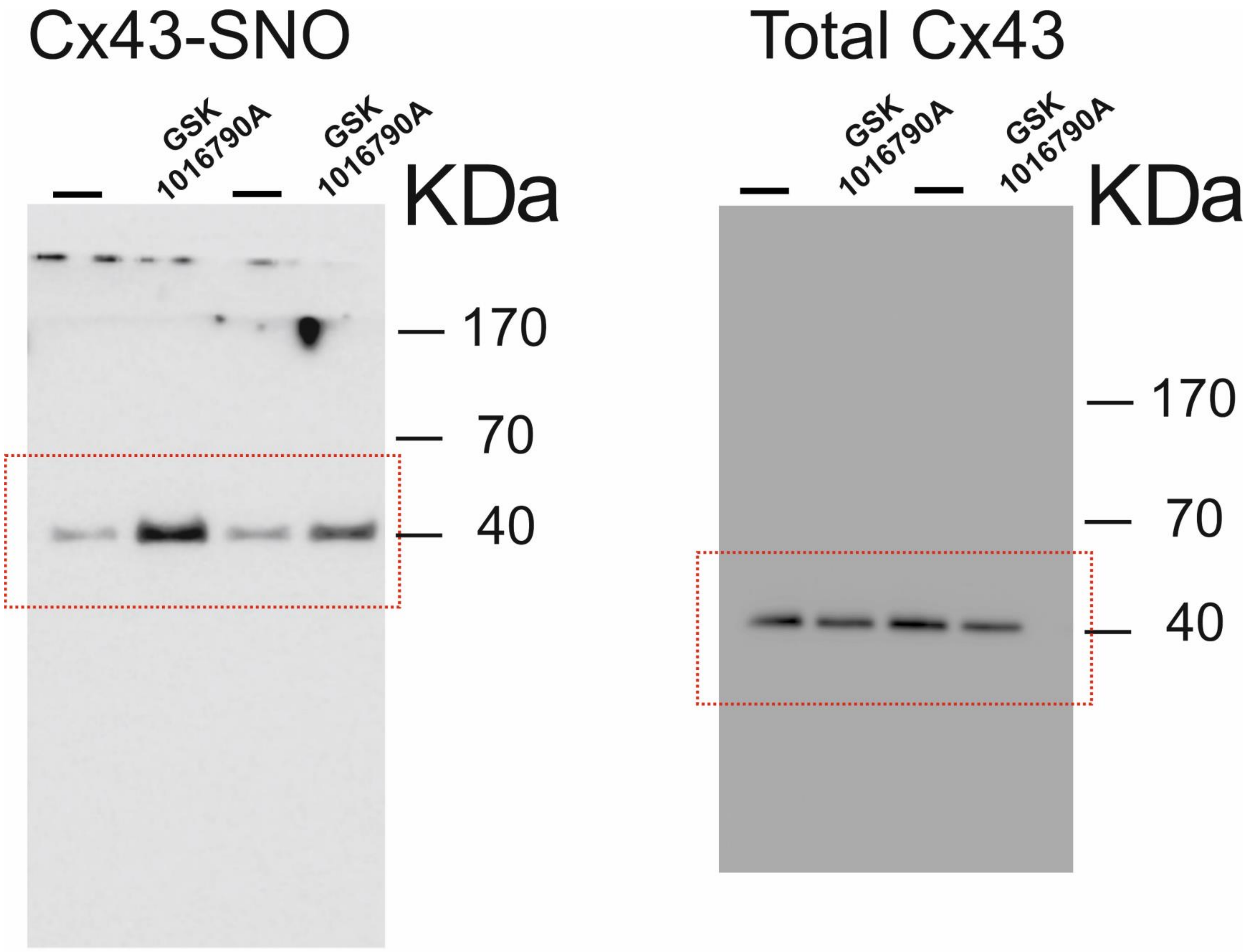
Cx43-SNO
—
GSK 1016790A
—
GSK 1016790A
KDa
170
70
40
Total Cx43
—
GSK 1016790A
—
GSK 1016790A
KDa
170
70
40
